# Pseudopatella Identified in Bilateral Gonarthrosis: A Case Report of Preoperative Recognition and Surgical Considerations in Total Knee Arthroplasty

**DOI:** 10.7759/cureus.90475

**Published:** 2025-08-19

**Authors:** Muhammed Yusuf Afacan, Mohammadreza Hajizadeh, Arın Celayir, Cumhur Deniz Davulcu, Nuri Aydin

**Affiliations:** 1 Department of Orthopaedics and Traumatology, Istanbul University-Cerrahpasa, Cerrahpasa Faculty of Medicine, Istanbul, TUR; 2 Department of Anatomy, Istanbul University-Cerrahpasa, Institute of Graduate Studies, Istanbul, TUR

**Keywords:** extensor mechanism, gonarthrosis, joint biomechanics, pseudopatella, total knee arthroplasty

## Abstract

Pseudopatella is a rare ossification within the quadriceps tendon, often associated with chronic mechanical stress or degenerative knee conditions, such as gonarthrosis. We report the case of a 59-year-old patient with bilateral gonarthrosis who underwent cruciate-retaining total knee arthroplasty (TKA). Intraoperatively, a pseudopatella was identified within the quadriceps tendon. To optimize the function of the extensor mechanism and prevent future complications, the ossified mass was carefully excised. The procedure proceeded with standard implant placement, ensuring proper alignment, stability, and soft tissue balance. Postoperative recovery was uneventful, and at follow-up, the patient achieved 100 degrees of flexion and full extension. This case underscores the importance of recognizing pseudopatella as a potential intraoperative finding during TKA, particularly in patients with advanced degenerative joint disease. Appropriate management can enhance joint biomechanics, reduce the risk of complications, and contribute to favorable functional outcomes.

## Introduction

Pseudopatella is a rare and atypical ossification within the quadriceps tendon, often arising in the context of chronic mechanical stress or degenerative changes in the knee joint, such as advanced gonarthrosis. Unlike the true patella - a natural sesamoid bone within the extensor mechanism - pseudopatella represents an acquired ossified structure [1]. Its formation is thought to result from repetitive strain, trauma, or altered biomechanics associated with joint degeneration. This uncommon condition is typically identified incidentally on radiological imaging or during surgery.

The clinical implications of a pseudopatella depend on its size, location, and impact on adjacent structures. While it may remain asymptomatic, larger or unfavorably positioned ossifications can disrupt the extensor mechanism, restrict joint mobility, or complicate surgical procedures, such as total knee arthroplasty (TKA) [2]. Accurate preoperative identification is critical for surgical planning and for anticipating intraoperative challenges.

Management of pseudopatella generally involves excision when it leads to functional impairment, causes pain, or interferes with surgical reconstruction. In the context of TKA, removal is often necessary to ensure proper soft tissue balance and optimal extensor mechanism function [3]. Careful dissection and preservation of the surrounding structures, particularly the patellar tendon, are essential to minimize complications and achieve favorable postoperative outcomes.

This case report describes the presence of a pseudopatella in a patient with bilateral gonarthrosis, emphasizing its preoperative recognition, intraoperative management during TKA, and the resulting functional recovery. It highlights the importance of awareness and appropriate treatment of this rare entity to facilitate successful surgical outcomes.

## Case presentation

**Figure 1:**
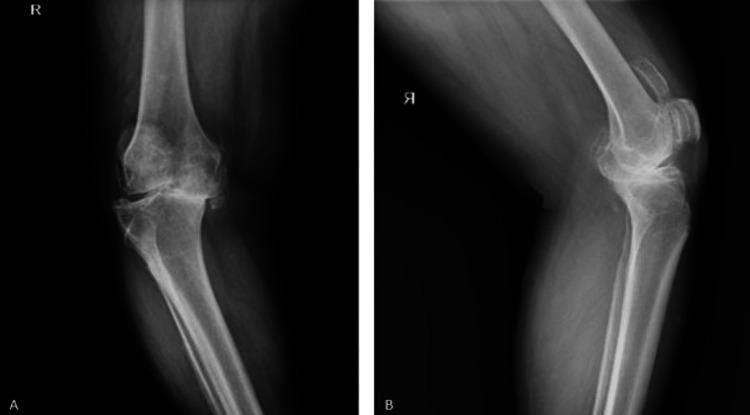
Preoperative radiological images of the patient (A) Anteroposterior (AP) view of the knee; (B) lateral view of the knee. Significant gonarthrosis and osteophytic changes are observed. The pseudopatella is visible in the lateral view.

A 59-year-old male patient presented with diffuse knee pain that had progressively worsened over the past several years, limiting his daily activities and ambulation. Informed consent was obtained prior to evaluation. Radiological imaging revealed bilateral advanced gonarthrosis (Figure 1). The patient had no known comorbidities, was not on any regular medication, and reported no history of previous surgeries or known drug allergies. His surgical and anesthetic history was unremarkable.

Physical examination showed crepitation in both knees, with flexion limited to 80 degrees and painful beyond that point, and extension limited to 10 degrees. The patient was scheduled for right cruciate-retaining TKA.

**Figure 2:**
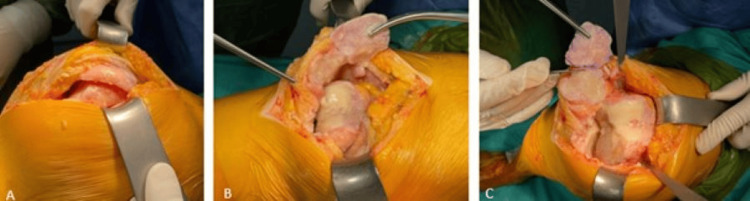
Intraoperative clinical images of the patient (A) Opening of the joint through a standard midline incision; (B) identification of the pseudopatella within the patellar tendon; (C) lateral retraction of the pseudopatella.

**Figure 3:**
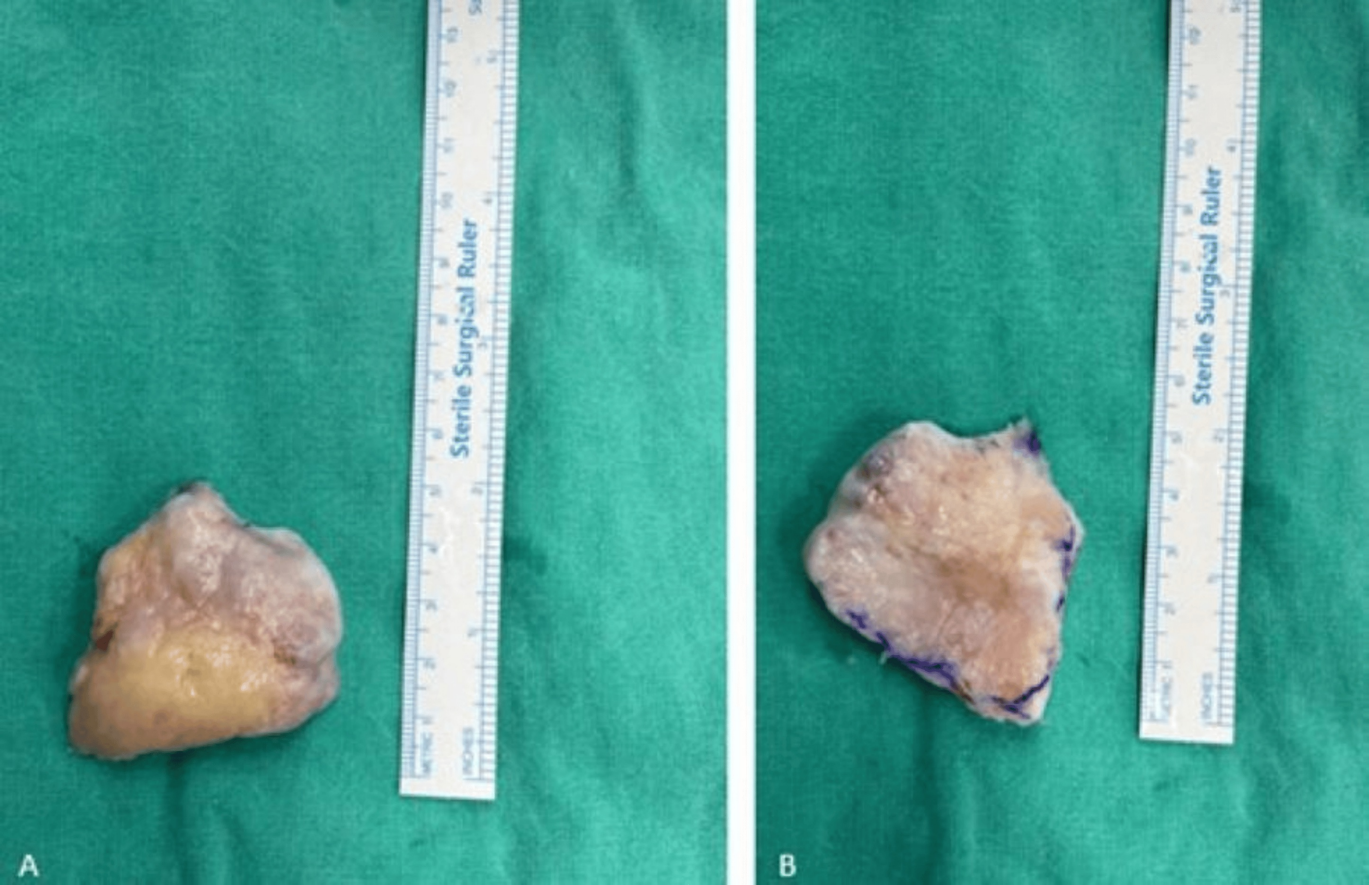
Post-excision images of the patient's pseudopatella (A) Anterior surface of the pseudopatella; (B) posterior surface of the pseudopatella.

During surgery, a standard midline incision and medial parapatellar arthrotomy provided joint exposure. Severe degenerative changes and osteophytes were noted. The femoral and tibial cuts were made using guides, and the surfaces were prepared for implants. A pseudopatella was identified in the patellar tendon and excised to restore optimal extensor mechanism function (Figures 2-3).

**Figure 4:**
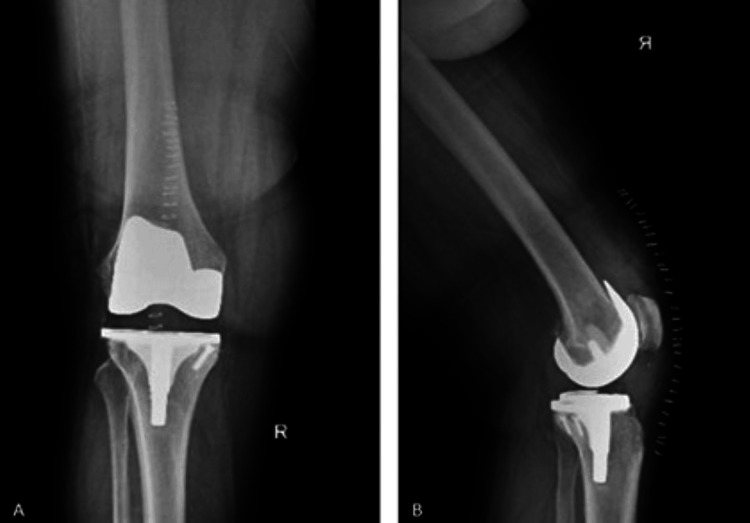
Postoperative radiological images of the patient (A) Anteroposterior (AP) view of the knee; (B) lateral view of the knee.

Trial components were placed, and alignment, stability, and soft tissue balance were confirmed before implanting the definitive components with bone cement. The wound was closed in layers after irrigation and hemostasis. Postoperative radiographs showed well-aligned implants, with no abnormalities (Figure 4).

The patient was mobilized with assistance from the physiotherapy team on postoperative day 1, initially using a walker for support. A structured rehabilitation protocol was initiated, including active-assisted range-of-motion exercises, quadriceps strengthening, and gait training. Pain was well controlled with multimodal analgesia, and the patient reported only mild discomfort during mobilization. Due to good pain tolerance and active participation in physiotherapy sessions, the patient demonstrated steady progress and was discharged on postoperative day 3. At the two-week follow-up, the surgical wound was healing without complication. Flexion had improved to 100 degrees, with full extension achieved, and there were no signs of extensor lag or instability.

## Discussion

Pseudopatella is a rare and poorly documented entity characterized by ossification within the quadriceps tendon. Although its exact etiology remains unclear, it is often associated with degenerative knee conditions such as gonarthrosis, where chronic mechanical stress, altered biomechanics, and local inflammation may contribute to its development [3]. In this case, the patient’s bilateral gonarthrosis, marked by advanced osteoarthritic changes and chronic joint degeneration, likely played a key role in the formation of the pseudopatella.

Preoperative detection of pseudopatella, as observed in our patient, is critical for surgical planning. Advanced imaging modalities, such as plain radiography or MRI, can identify ossifications within the extensor mechanism and help assess their impact on knee biomechanics. In this case, the pseudopatella was clearly visualized on the lateral radiograph, allowing us to anticipate its implications during TKA.

The intraoperative management of pseudopatella presents unique challenges. If left unaddressed, the ossified structure may impair extensor mechanism function, disrupt soft tissue balance, and compromise TKA outcomes [4]. In our patient, the decision to excise the pseudopatella was made to optimize the alignment and functionality of the extensor mechanism and to ensure appropriate implant positioning. Particular care was taken to preserve the patellar tendon during excision, maintaining the continuity of the extensor apparatus.

There is limited literature specifically addressing the impact of pseudopatella on TKA. While previous reports have described conditions such as patella baja, quadriceps fibrosis, or patellar hypoplasia in arthritic knees, few have explicitly documented pseudopatella and its postoperative implications for extensor function [5]. Our case contributes to the growing body of evidence that emphasizes the importance of recognizing rare extensor mechanism anomalies during TKA [6]. Moreover, it reinforces that excision of such ossifications - when performed with surgical precision - does not compromise extensor continuity and may, in fact, improve implant alignment and soft tissue balancing.

Postoperatively, our patient experienced a favorable outcome, with no wound complications and an improved range of motion, including 100 degrees of knee flexion and full extension. This highlights the importance of intraoperative management of pseudopatella, as failure to address such anomalies may lead to residual functional deficits or postoperative complications.

This case supports previous reports of successful surgical outcomes in patients with pseudopatella, without postoperative extensor lag or stiffness. In contrast to earlier descriptions of complications - such as patellar tendon rupture, poor patellar tracking, or persistent anterior knee pain - our patient’s recovery demonstrates that timely recognition and appropriate surgical management can lead to optimal results, even in the presence of rare anatomical variations [7].

Furthermore, this case underscores the importance of comprehensive surgical planning when encountering unexpected intraoperative findings. Orthopedic surgeons should remain vigilant for atypical ossifications, particularly in patients with advanced degenerative joint disease undergoing TKA. Thorough preoperative imaging and functional assessment are essential to guide intraoperative decisions and optimize surgical outcomes.

Although pseudopatella is rare, its recognition and appropriate management are essential for achieving favorable functional outcomes following TKA [8]. Further studies and additional case reports are warranted to improve our understanding of its pathophysiology, clinical prevalence, and long-term implications. Standardized protocols for its evaluation and treatment may ultimately help optimize care for patients presenting with this unusual finding. This case adds to the limited literature on pseudopatella and highlights the value of preoperative awareness and meticulous intraoperative technique in achieving successful outcomes.

## Conclusions

Pseudopatella, though a rare and often overlooked entity, can pose significant challenges during TKA, particularly in patients with advanced gonarthrosis. Its presence within the quadriceps tendon can disrupt extensor mechanism function, compromise soft tissue balance, and interfere with proper implant placement. This case underscores the critical role of thorough preoperative imaging and surgical preparedness in identifying and addressing such atypical findings. Surgical excision of the pseudopatella, when indicated, can restore normal biomechanics and contribute to optimal postoperative recovery. Awareness of this condition and its implications is essential for orthopedic surgeons to ensure precise intraoperative management and to achieve favorable functional outcomes following TKA.
